# *Chlamydia trachomatis* Infection and Its Association with Human Papillomavirus Among Adolescent Girls and Young Women of Eastern Cape, South Africa

**DOI:** 10.3390/epidemiologia6040079

**Published:** 2025-11-12

**Authors:** Olufunmilayo O. Akapo, Zizipho Z. A. Mbulawa

**Affiliations:** 1 Department of Laboratory Medicine and Pathology, Walter Sisulu University, Mthatha 5100, South Africa; oakapo@wsu.ac.za; 2 National Health Laboratory Service, Nelson Mandela Academic Hospital, Mthatha 5100, South Africa

**Keywords:** sexually transmitted infections, *Chlamydia trachomatis*, human papillomavirus, HPV vaccines, adolescent girls and young women, coinfection

## Abstract

Background/Objectives: Adolescent girls and young women (AGYW) have a high burden of sexually transmitted infections (STIs). This study examined the prevalence of *Chlamydia trachomatis*, its association with human papillomavirus (HPV), and other associated factors among AGYW of rural communities. Methods: Secondary data on *C. trachomatis*, HPV, and linked questionnaires from 214 sexually active AGYW were used. Self-collected vaginal specimens were previously tested using the Allplex™ STI Essential Assay and Roche Linear Array HPV genotyping assay. Results: The overall prevalence of *C. trachomatis* was 29.4% (63/214), and it was not influenced by age. The majority of the AGYW were *C. trachomatis* negative and HPV positive (52.4%, 111/212), followed by being *C. trachomatis* and HPV co-infected (23.6%, 50/212), *C. trachomatis* and HPV co-negative (18.4%, 39/212) and least were *C. trachomatis* positive and HPV negative (5.7%, 12/212). There was an increased prevalence of being HPV infected among *C. trachomatis* individuals than being *C. trachomatis* positive among HPV positive individuals (RR: 2.60, 95% CI: 2.00–3.38, *p* < 0.0001). *C. trachomatis* positive AGYW had a significantly higher association of HPV types targeted by Cervarix^®^ HPV vaccine (HPV-16 and/or -18) than *C. trachomatis* negatives (RR: 2.58, 95% CI: 1.37–4.82, *p* = 0.005), targeted by Gardasil^®^4 HPV vaccine (HPV-6, -11, -16 and/or -18; RR: 2.21, 95% CI: 1.32–3.65, *p* = 0.005) and Gardasil^®^9 HPV vaccine (HPV-6, -11, -16, -18, -31, -33, -45, -52 and/or -58; RR: 1.92, 95% CI: 1.37–2.67, *p* < 0.001). Conclusions: There was a high burden of *C. trachomatis* and HPV coinfection. *C. trachomatis* coinfection influenced HPV genotype prevalence and distribution, including those that are targeted by the current commercial HPV vaccines, suggesting that the high burden of *C. trachomatis* among AGYW may pose challenges to the ongoing HPV vaccination program. Integrated STI screening and prevention strategies are needed in rural South African settings.

## 1. Introduction

### 1.1. Global and Regional Burden of STIs

Sexually transmitted infections (STIs) continue to pose a significant global public health challenge, with over one million new infections occurring daily worldwide [1,2]. Among these, *Chlamydia trachomatis* is one of the most common bacterial STIs globally, while human papillomavirus (HPV) is the most prevalent viral STI [2]. The burden of these infections is particularly high among adolescent girls and young women (AGYW). In South Africa, the prevalence of *C. trachomatis* and HPV among AGYW has been reported high as 41.6% and 85.0%, respectively [3,4,5,6,7]. Notably, in sub-Saharan Africa, the prevalence of chlamydia increased by 34.5% from 2010 to 2020 [1], underscoring the growing public health concern in the region despite *C. trachomatis* being a curable infection [4].

### 1.2. STI Management Approaches

In many low-resource settings, including South Africa, syndromic management is the predominant strategy for STI diagnosis and treatment due to the high cost, limited infrastructure, and shortage of skilled personnel required for molecular diagnostic methods [8]. Unfortunately, this approach is inadequate given the high rate of asymptomatic infections, particularly with *C. trachomatis* and HPV [7,9,10]. For example, many AGYW may not present with overt symptoms, leading to missed diagnoses and untreated infections. In contrast, HPV molecular testing has recently been integrated into cervical cancer screening programs in both public and private healthcare sectors in South Africa, often used as a reflex test before cytology [11].

### 1.3. C. trachomatis and HPV Coinfections and Interactions

**Figure 1:**
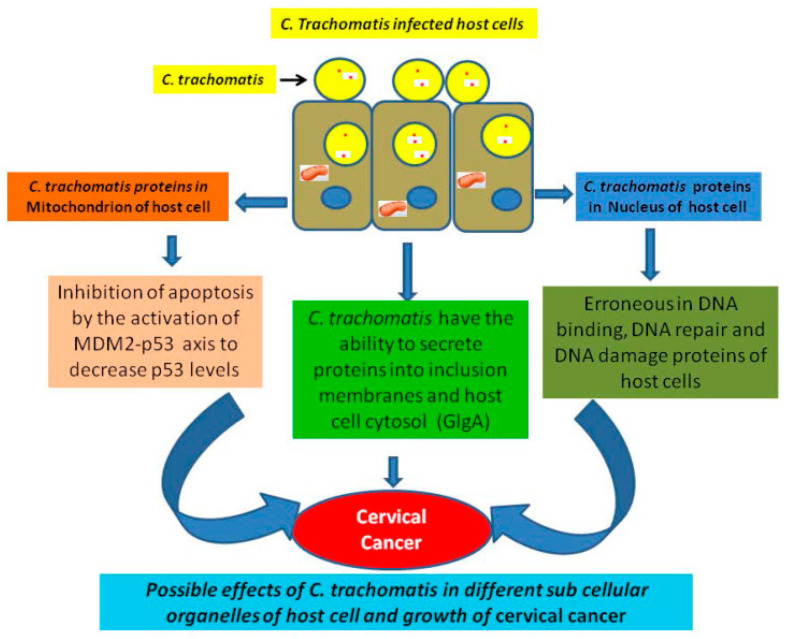
Mechanism pathway of *C. trachomatis* leading to cervical cancer [18].

Coinfection with *C. trachomatis* and other STIs such as HPV, HIV, and herpes simplex virus (HSV) is common and clinically significant. These coinfections can exacerbate disease progression, complicate treatment, and undermine prevention strategies. Specifically, *C. trachomatis* has been implicated as a cofactor in the persistence of HPV infections and the subsequent development of cervical cancer [12,13,14,15,16]. Biological interactions, including protein–protein interactions between *C. trachomatis* and HPV, may facilitate viral persistence and oncogenesis [14,15]. *C. trachomatis* infection induces a pro-inflammatory environment in the genital tract, characterized by the recruitment of immune cells and elevated cytokine production [15,16]. This inflammatory milieu may enhance HPV acquisition and persistence by increasing the availability of susceptible target cells. In addition, *C. trachomatis* is known to cause epithelial disruption and microabrasions, which can facilitate HPV entry into basal epithelial layers, a prerequisite for viral infection and persistence [13,14]. Beyond structural damage, immune modulation represents another critical pathway. *C. trachomatis* has been shown to interfere with antigen presentation and adaptive immune responses, dampening local immunity [13,17]. Protein–protein interactions between *C. trachomatis* and HPV-related host proteins may further dysregulate cellular pathways involved in apoptosis, DNA repair, and oncogenesis, thereby enhancing the likelihood of HPV persistence and malignant progression (Figure 1) [14,15,18].

### 1.4. Prevention and Control Strategies

Currently, no vaccine exists for *C. trachomatis*, highlighting the urgent need for enhanced prevention, screening, and treatment interventions [19]. Conversely, several prophylactic HPV vaccines (e.g., Cervarix^®^, Gardasil^®^4, and Gardasil^®^9) are available, and some have been incorporated into the South African national vaccination program since 2014. This program targets girls aged nine and older in Grade 4 of public schools and 1 uses a two-dose schedule of the Cervarix^®^ vaccine [20,21]. *C. trachomatis* coinfection has been linked to variations in HPV genotype distribution, including those genotypes targeted by current vaccines; the high prevalence of *C. trachomatis* and its coinfection with HPV may challenge the HPV prevention strategies [13,22].

### 1.5. Study Rationale

Given the significant prevalence of both *C. trachomatis* and HPV among AGYW in South Africa and their potential for coinfection and interaction, there is a pressing need for localized data to inform public health strategies. Existing research has highlighted a gap in data on the prevalence and impact of *C. trachomatis* on HPV infection and genotype distribution, particularly among unvaccinated AGYW in the Eastern Cape Province. This study addresses this gap by examining the prevalence of *C. trachomatis*, its influence on HPV infection and genotype distribution, and associated risk factors among sexually active AGYW in rural Eastern Cape communities. Such data are essential for informing STI prevention programs and HPV vaccine effectiveness monitoring.

## 2. Materials and Methods

### 2.1. Study Design and Participants

This was a retrospective cross-sectional analysis based on data collected from adolescent girls and young women (AGYW) who participated in the HPV Education Intervention Study [5]. Participants were recruited between April and May 2019 from two no-fee public high schools in the Chris Hani District Municipality, Eastern Cape Province, South Africa. All study procedures, including sample collection and questionnaire administration, were conducted in nearby primary healthcare facilities. Data were retrospectively collected if the participants were females aged 15–24 years, self-reported a history of vaginal sexual activity, had valid laboratory results for *Chlamydia trachomatis* and/or HPV testing, and completed the linked closed-ended questionnaire assessing sociodemographic and behavioral variables. A total of 214 AGYW with complete and valid data were included in this analysis.

### 2.2. Data Collection and Laboratory Testing

The parent study collected data on demographics (age, school grade), sexual and reproductive health behaviors (age at sexual debut, number of sexual partners, history of pregnancy, condom use), and lifestyle factors (alcohol use, smoking) through closed-ended questionnaires. HIV status was confirmed by rapid diagnostic testing conducted by trained healthcare personnel at the time of recruitment. However, 67 of the study participants were not tested for HIV. Self-collected vaginal specimens were collected using Evalyn^®^ Brush (Rovers^®^ Medical Devices B.V., Oss, The Netherlands) according to the manufacturer’s standardized instructions. Nucleic acid was extracted using the MagNA Pure Compact System (Roche Molecular Systems, Inc., Branchburg, NJ, USA) and the MagNA Pure Compact Nucleic Acid Isolation Kit. The presence of *C. trachomatis* was detected using the Allplex™ STI Essential Assay (Seegene Inc., Seoul, Republic of Korea), a multiplex real-time PCR test, according to the manufacturer’s instructions. Amplification and analysis were conducted on the CFX96™ Real-Time PCR Detection System (Bio-Rad, Hercules, CA, USA) using CFX Manager™ IVD v1.6 software and Seegene Viewer Software version 3.3 for result interpretation. The HPV genotyping was performed using the Roche Linear Array HPV Genotyping Test (Roche Molecular Systems, Inc., Branchburg, NJ, USA), which detects 37 HPV genotypes, including high-risk and low-risk types. Genotypes included HPV-6, -11, -16, -18, -26, -31, -33, -35, -39, -40, -42, -44, -45, -51, -52, -53, -54, -55, -56, -58, -59, -61, -62, -64, -66, -67, -68, -69, -70, -71, -72, -73, -81, -82, -83, -84, -89, and -IS39. A total of 214 AGYW with valid *C. trachomatis* and/or HPV data were retrospectively recruited from the main study reported by Onywera et al. and Mbulawa et al. [5,23], respectively.

### 2.3. Statistical Analysis

Data was coded and captured in Microsoft Excel 2016 (Microsoft Corporation, Seattle, WA, USA). GraphPad Prism software v8.0.1. was used to perform all statistical analyses. Descriptive statistics (median and interquartile range (IQR)) were used to describe the data. Fisher’s exact and the relative risk (RR) were used to compare the variables between groups. RRs with their corresponding 95% Confidence Interval (CIs) and *p*-values were computed and tabulated. Statistical significance was set at *p* value ≤ 0.05.

## 3. Results

### 3.1. Chlamydia Trachomatis Prevalence and Associated Factors Among AGYW

**Figure 2:**
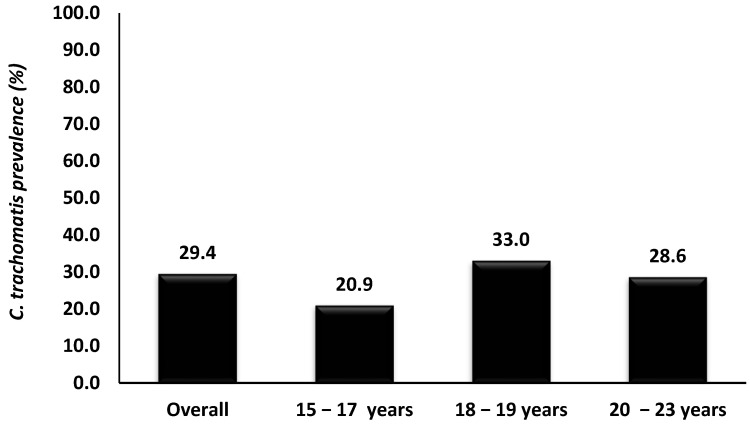
*Chlamydia trachomatis* prevalence according to age among adolescent girls and young women of Eastern Cape Province, South Africa.

The study participants’ characteristics have been previously described elsewhere [5,21]. The overall prevalence of *Chlamydia trachomatis* among AGYW in the Eastern Cape Province was 29.4% (63/214, Figure 2). No significant associations were observed between *C. trachomatis* prevalence and the demographic or behavioral factors assessed, including age, HIV status, or sexual behaviors (Table 1).

### 3.2. Chlamydia trachomatis and HPV Infection Among AGYW

**Figure 3:**
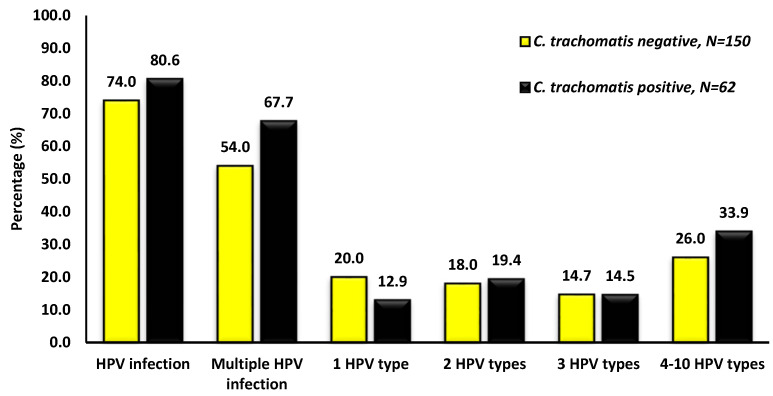
Prevalence of human papillomavirus (HPV) among *Chlamydia trachomatis* positive and negative among adolescent girls and young women of Eastern Cape Province, South Africa.

Among AGYW, the overall HPV prevalence was 79.9% (161/212). The most frequent infection pattern was HPV positivity without *C. trachomatis* (52.4%), followed by coinfection (23.6%), and a smaller proportion were co-negative (18.4%) or *C. trachomatis*-positive but HPV-negative (5.7%). Multiple HPV infections were more common among *C. trachomatis*-positive AGYW (67.7%) compared to *C. trachomatis*-negative AGYW (54.0%). Detailed distributions of HPV and *C. trachomatis* co-infection patterns are presented in Figure 3.

**Figure 4:**
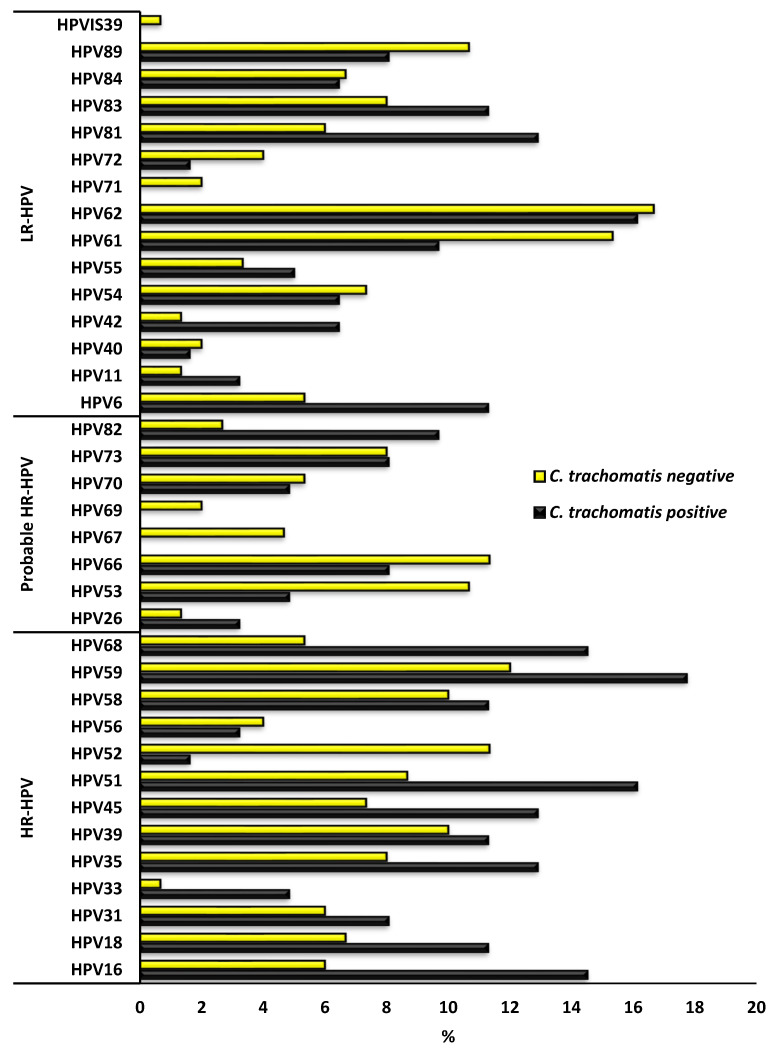
Prevalence of human papillomavirus (HPV) genotype distribution among *Chlamydia trachomatis* positive and negative adolescent girls and young women of Eastern Cape Province, South Africa.

**Figure 5:**
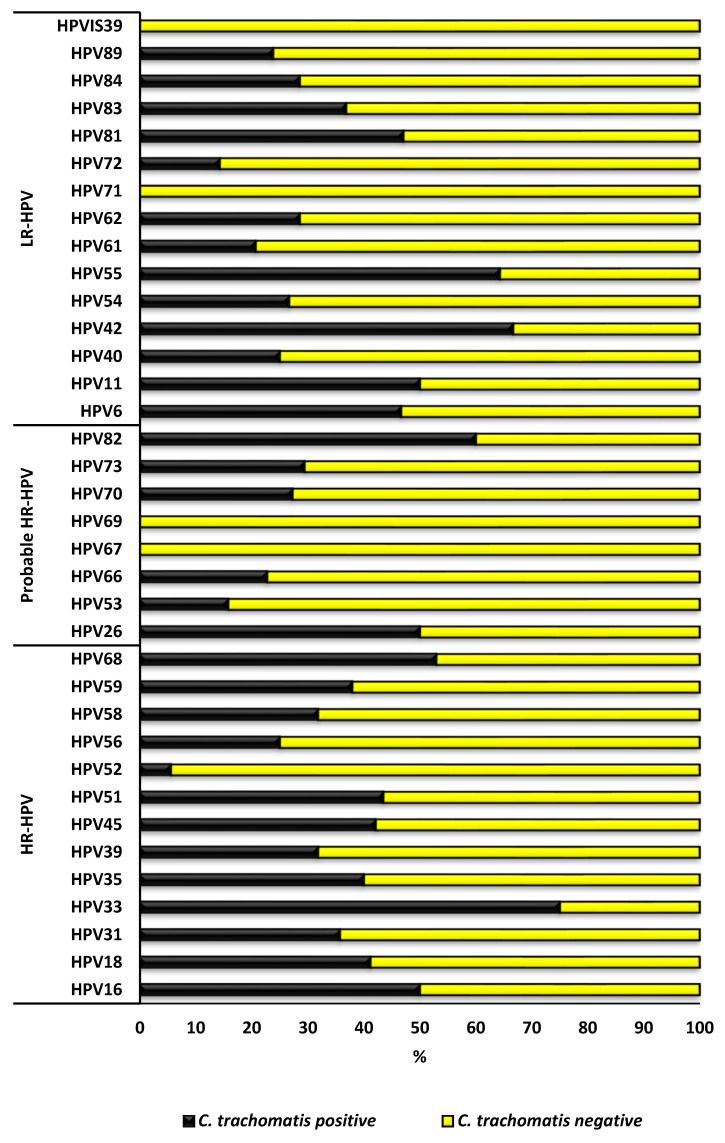
Human papillomavirus (HPV) genotype distribution among the HPV positives that were *Chlamydia trachomatis* positive or negative adolescent girls and young women of Eastern Cape Province, South Africa.

The overall HPV genotype distribution according to *C. trachomatis* positive and negative status is presented in Figure 4. The prevalence of the majority of HR-HPV types was higher among *C. trachomatis*-positive AGYW than among *C. trachomatis*-negative AGYW; specifically, HPV-16, -18, -31, -33, -35, -39, -45, -51, -58, -59, and -68, except for HPV-52 and -56. At the same time, most of the LR-HPV and probable HR-HPV types prevalence was higher among *C. trachomatis*-negative than the *C. trachomatis*-positive AGYW; specifically, HPV-40, -53, -54, -61, -62, -66, -80, -72, -84 and -89, except HPV-6, -11, -26, -42, -55, -73, -81, -82 and -83. Figure 5 presents the HPV genotype distribution among the HPV positive AGYW who were *Chlamydia trachomatis* positive or negative. None of the factors presented in Table 1 were associated with *C. trachomatis* and HPV coinfection among AGYW.

### 3.3. Association of Chlamydia trachomatis with HPV Types Targeted by Current HPV Vaccines

*Chlamydia trachomatis*-positive AGYW had significantly higher prevalence of HPV types covered by Cervarix^®^, Gardasil^®^4, and Gardasil^®^9 vaccines than *C. trachomatis* negatives. The current HPV commercial vaccines do not cover HPV-35; however, due to its high burden in women of African ancestry and its role in accounting for significant cases of cervical cancer, they were included in this analysis. *C. trachomatis* positive AGYW had 61% higher burden of HPV-35 than *C. trachomatis* negative individuals even though not statistically significant (Table 2).

## 4. Discussion

This study provides novel insights into the prevalence of *Chlamydia trachomatis* and its interaction with human papillomavirus (HPV) among adolescent girls and young women (AGYW) in the Eastern Cape Province of South Africa. Overall, the prevalence of *C. trachomatis* was 29.4%, and HPV prevalence reached 79.9%. Nearly one-quarter (23.6%) of participants were coinfected with both pathogens. A high rate of HPV and *C. trachomatis* coinfection among AGYW was observed in a predominantly HIV-negative population, as less than five percent of the study population were HIV-positive; this increases the chances of HIV acquisition in the future [15,16]. This small number of HIV-positive individuals limited the ability to fully explore its effect on *C. trachomatis* and HPV coinfection. Given that HIV infection is known to modulate immune function and increase susceptibility to both *C. trachomatis* and HPV acquisition and persistence, residual confounding may remain, particularly for coinfection dynamics. Future studies with larger HIV-positive subgroups will be necessary to clarify the independent and interactive effects of HIV on STI coinfection patterns.

The findings from this study reinforce the high burden of STIs in this population, consistent with previous South African and sub-Saharan African studies reporting similarly elevated levels of curable STIs and HPV infection [3,4,5,6,7,24]. The observed *C. trachomatis* prevalence (29.4%) is higher than reported in some South African provinces, such as Gauteng (approximately 18%), but lower than the Western Cape, where prevalence exceeded 40% in AGYW [7,24]. In the current study, there was an increased risk of being HPV infected among *C. trachomatis*-infected individuals than of being *C. trachomatis*-positive among HPV positive individuals. HPV is reported to have an impact on the pathogenesis of *C. trachomatis* [17,24]. The prevalence in this current study is also considerably higher than that reported among pregnant women in Durban, KwaZulu-Natal, where *C. trachomatis* prevalence was 11% [25] and STI prevalence levels of ~13% observed among women in general cohorts in the same province [26]. Similarly, the prevalence of HPV in our study was 79.9% (161/212), which far exceeds estimates from women attending reproductive health clinics in Nairobi, Kenya (31–36%) [27], and from general populations in rural Malawi (~20%) [28,29]. These comparisons reinforce that AGYW in the Eastern Cape face a disproportionately high burden of both *C. trachomatis* and HPV relative to women in other South African provinces and similar sub-Saharan African settings. HPV prevalence was strikingly high (79.9%), aligning with previous findings of HPV rates exceeding 80% among South African adolescents [5]. This persistent burden of infection highlights a critical unmet need for effective STI prevention, screening, and treatment strategies in rural South African contexts.

Coinfection with *C. trachomatis* and HPV was common (23.6%), and multiple HPV infections were more frequent among *C. trachomatis*-positive AGYW (67.7%) compared with their *C. trachomatis*-negative counterparts (54.0%). Conversely, the likelihood of being *C. trachomatis* positive was lower among HPV-infected individuals. This asymmetry suggests that *C. trachomatis* may play a facilitating role in HPV acquisition or persistence, consistent with previous findings [23,24]. Although these trends should not be interpreted as causal associations, they raise concerns about the role of *C. trachomatis* in shaping HPV persistence and genotype distribution. Laboratory and epidemiological studies have suggested that *C. trachomatis* may act as a cofactor for HPV persistence and cervical carcinogenesis by modulating local immune responses or through protein–protein interactions with HPV [13,14,15,24]. Our data showing higher proportions of high-risk HPV genotypes (e.g., HPV-16, -18, -31, -33, and -45) in coinfected individuals are consistent with these hypotheses, although longitudinal studies are needed to establish temporal relationships. The bidirectional impact of HR-HPV and *C. trachomatis* infection likely reflects several biological mechanisms that act synergistically to promote cervical disease progression (Figure 1). *C. trachomatis* infection induces chronic inflammation, recruitment of immune cells, and production of reactive oxygen species, which can cause epithelial damage and genomic instability that facilitate HPV persistence. Additionally, *C. trachomatis* can disrupt epithelial barriers and modulate cytokine signaling, thereby increasing susceptibility to HPV entry. Conversely, HPV oncoproteins such as E6 and E7, impair local immune surveillance and epithelial repair mechanisms, creating a microenvironment favorable to *C. trachomatis* persistence. Together, these processes contribute to prolonged HPV infection, increased multiplicity of high-risk HPV types, and a higher likelihood of progression toward cervical intraepithelial neoplasia and cancer.

Importantly, this high coinfection rate was observed in a population where fewer than 5% of participants self-reported as HIV-positive. Not all study participants were tested, which may underestimate the true HIV prevalence of the study population. This is relevant because HIV-related immune dysregulation can alter both HPV persistence and *C. trachomatis* susceptibility, potentially confounding observed coinfection dynamics [30,31]. Therefore, our findings regarding the interaction between HPV and *C. trachomatis* should be interpreted with caution in the context of HIV as a possible unmeasured confounder. *C. trachomatis*-positive AGYW had a higher prevalence of HPV types targeted by existing vaccines and some of the epidemiologically important HR-HPV types. For instance, the association of HPV-16 and/or -18 (Cervarix^®^ coverage) was more than two-fold higher in coinfected individuals (25.8% vs. 10.0%). Similar patterns were observed for Gardasil^®^4 (33.9% vs. 15.3%) and Gardasil^®^9 (56.5% vs. 29.3%) vaccine types. These findings highlight potential challenges for HPV vaccine impact in populations with a high burden of *C. trachomatis*. While HPV vaccination has demonstrated strong efficacy in reducing vaccine-type infections, coinfections with *C. trachomatis* may influence genotype distribution and persistence, possibly complicating vaccine monitoring efforts [20,21,22].

### 4.1. Clinical and Public Health Implications

The lack of significant associations between *C. trachomatis* prevalence and demographic or behavioral risk factors in this study suggests that the infection association is widespread in this setting and not easily predicted by self-reported characteristics. This finding supports calls for universal or routine screening approaches, rather than association-based testing, especially in high-burden communities [8,10]. Moreover, the predominance of asymptomatic infections makes reliance on syndromic management inadequate for both *C. trachomatis* and HPV detection [9,10]. Integration of molecular-based STI testing into existing reproductive health services could improve case detection and prevent long-term reproductive complications.

### 4.2. Strengths and Limitations

This study benefits from the use of molecular diagnostic assays, which provide robust detection of both *C. trachomatis* and HPV infections, and from the inclusion of detailed behavioral and demographic data. However, certain limitations should be acknowledged. First, the retrospective design restricts causal inference, and observed trends should not be considered definitive associations. Second, the use of self-reported behavioral data may be subject to recall or social desirability bias, which means that some of the behaviors may be underestimated or overestimated. The use of self-collected vaginal specimens in this study provided a practical and acceptable method for sample acquisition among AGYW; it may also introduce potential sources of bias. Inconsistent adherence to collection instructions could have resulted in variable specimen quality, potentially leading to under-detection of *Chlamydia trachomatis* or HPV in some participants. However, previous studies have demonstrated that self-collected specimens perform comparably to clinician-collected samples when processed using molecular assays, suggesting that any underestimation is likely modest rather than systematic. Third, the study population was drawn from a limited geographic region, which may limit generalizability to all AGYW in South Africa.

Fourth, the modest sample size (*n* = 214) of this study may have limited statistical power, which could have reduced the ability to detect associations of small to moderate effect. Additionally, the study population was relatively homogeneous, with most participants drawn from similar age groups, school environments, and socioeconomic contexts, which may have limited variability in risk factors. While the sample size was sufficient to provide robust prevalence estimates for *C. trachomatis* and HPV, it may not have been adequately powered to detect more nuanced relationships between behavioral risk factors and infection status. Future studies with larger and more diverse populations, coupled with the triangulation of self-reported data with biological or partner-reported measures, will be important to clarify the role of behavioral and demographic determinants in shaping infection risk. Despite these limitations, the data generated are valuable for informing public health initiatives.

### 4.3. Policy Implications and Future Studies

The high prevalence of *C. trachomatis* and HPV coinfection observed in this study has direct implications for reproductive health services, particularly in rural and underserved populations where access to diagnostic testing and preventive care remains limited. The reproductive health consequences of untreated *C. trachomatis,* including pelvic inflammatory disease, infertility, and adverse pregnancy outcomes, combined with the oncogenic potential of persistent HPV infection, underscore the need for integrated service delivery. Incorporating *C. trachomatis* testing into routine reproductive health care, especially alongside cervical cancer prevention programs, could substantially strengthen the effectiveness of existing services.

The high prevalence of *Chlamydia trachomatis* suggests that current reliance on syndromic management is insufficient to address the accurate scale of asymptomatic infections among AGYW. Policymakers should therefore consider the targeted interventions, including routine *C. trachomatis* testing incorporated into adolescent health services and delivered in tandem with the national HPV vaccination program, targeted education for AGYW, their caregivers, and community stakeholders is essential to improve awareness of asymptomatic STIs, coinfection association and the importance of HPV vaccination. Programs should leverage peer education, digital health platforms, and school-based initiatives to ensure broad reach and engagement. This strategy would enable early detection and treatment of bacterial STIs while simultaneously preventing HPV-related disease, thereby addressing both pathogens synergistically. The policy implications of these results are substantial. Health education programs should address both viral and bacterial STI risks, emphasizing condom use, healthcare-seeking behavior, and vaccine uptake [32,33]. Linking HPV vaccination delivery with broader reproductive health services could improve program efficiency and mitigate the combined burden of HPV and *C. trachomatis* among AGYW. These data argue for scaling up adolescent-friendly STI screening services, integrating *C. trachomatis* testing with HPV vaccination programs, and enhancing community-based health education to address persistent gaps in awareness and care.

Future studies should adopt longitudinal designs to examine the temporal relationship between *C. trachomatis* and HPV persistence, as well as the potential modifying role of coinfection on vaccine effectiveness. Expanding research to include larger, more diverse populations across South Africa would also strengthen the evidence base for integrated STI prevention and vaccination strategies. Future studies that directly compare prevalence and coinfection dynamics across multiple South African provinces and countries will help refine intervention strategies, ensuring they are both locally relevant and globally informed. By situating findings of this study within the broader landscape, this study strengthens the evidence base for integrated STI control and adolescent sexual health programs in resource-limited settings worldwide.

## 5. Conclusions

This study reveals a high prevalence of *Chlamydia trachomatis* and HPV, as well as notable coinfection patterns, among adolescent girls and young women in the Eastern Cape Province. The observed burden of *C. trachomatis* (29.4%) and HPV (79.9%) underscore the ongoing challenge of STI prevention and control in this setting. Importantly, coinfection was associated with a higher prevalence of multiple HPV infections and vaccine-targeted HPV genotypes, highlighting potential implications for long-term cervical cancer prevention. These findings contribute valuable evidence from the Eastern Cape, a province that is often underrepresented in epidemiological surveillance compared to Gauteng and the Western Cape. While the prevalence estimates in this study fall between those previously reported in these two provinces. The results emphasize that the STI epidemic is not confined to urban centers and may manifest differently across diverse South African provinces. Extending such research beyond traditionally studied regions is critical for tailoring interventions that address context-specific drivers of infection. Moreover, when placed in a global context, the findings resonate with reports from other low-resource, high-burden regions in sub-Saharan Africa and comparable settings in Asia and Latin America, where adolescent girls face overlapping vulnerabilities related to limited healthcare access, high rates of asymptomatic infection, and constrained preventive infrastructures. This contextualization underscores the broader applicability of our results, demonstrating that strategies integrating molecular diagnostics, routine screening, and HPV vaccination must be prioritized not only in South Africa but also in similar socioeconomic and geographic contexts. This work fills a critical knowledge gap by providing the first detailed assessment of *C. trachomatis* prevalence, coinfection patterns, and implications for HPV vaccination in the Eastern Cape. The data generated from this study will also contribute to the existing knowledge on *C. trachomatis* and its co-infection with HPV among AGYW in the Eastern Cape Province.

## Figures and Tables

**Table 1:** Factors associated with *Chlamydia trachomatis* prevalence among AGYW of Eastern Cape Province, South Africa.

Variable	*C. trachomatis* n/N (%)	RR (95% CI)	*p*-Value
**Age**			
15–17 years	9/43 (21.0)	Ref	
18–19 years	38/115 (33.0)	1.86 (0.87–3.03)	0.172
20–23 years	16/56 (28.6)	1.37 (0.69–2.79)	0.486
**Sexual debut age**			
≤15 years	24/85 (28.2)	Ref	
16–17 years	38/128 (29.7)	1.05 (0.69–1.63)	0.878
**HIV status**			
Positive	3/7 (42.9)	Ref	
Negative	44/140 (31.4)	0.73 (0.39–2.04)	0.680
Not tested	16/67 (23.9)	0.56 (0.26–1.62)	0.363
**Education**			
Grade 8	4/12 (33.3)	Ref	
Grade 9	7/16 (43.8)	1.31 (0.53–3.57)	0.705
Grade 10	15/55 (27.3)	0.82 (0.37–2.14)	0.729
Grade 11	16/65 (24.6)	0.74 (0.34–1.93)	0.500
Grade 12	21/64 (32.8)	0.98 (0.47–2.51)	>0.999
**Ever consumed alcohol**			
No	11/39 (28.2)	Ref	
Yes	52/175 (29.7)	1.05 (0.64–1.87)	>0.999
**Number of lifetime sexual partners**			
1	13/50 (26.0)	Ref	
2	27/79 (34.2)	1.32 (0.77–2.33)	0.435
≥3	21/71 (29.6)	1.14 (0.64–2.01)	0.688
**Number of new sexual partners past 3-months**			
0	8/32 (25.0)	Ref	
1	46/142 (32.4)	1.30 (0.73–2.54)	0.527
≥2	7/26 (26.9)	1.08 (0.45–2.51)	>0.999
**Ever pregnant**			
No	46/167(27.5)	Ref	
Yes	17/47 (36.2)	1.31 (0.82–2.01)	0.279
**Vaginal discharge/itching**			
No	24/76 (31.6)	Ref	
Yes, 1-month	17/66 (26.8)	0.82 (0.48–1.37)	0.464
Yes, >1 months	22/72 (30.6)	0.97 (0.60–1.56)	>0.999
**Genital Warts/Blisters**			
No	47/168 (28.0)	Ref	
Yes, 1-month	9/29 (31.0)	1.11 (0.59–1.89)	0.824
Yes, >1 months	6/13 (46.2)	1.65 (0.80–2.77)	0.206

**Table 2:** Association of *Chlamydia trachomatis* with the prevalence of HPV types targeted by current HPV vaccines.

*C. trachomatis* Status	HPV Vaccine/Type	n	%	RR (95% CI)	*p*-Value
*C. trachomatis* negative, N = 150	Cervarix^®^	15	10.0	Ref	
*C. trachomatis* positive, N = 62	Cervarix^®^	16	25.8	2.58 (1.37–4.82)	**0.005**
					
*C. trachomatis* negative, N = 150	Gardasil4^®^	23	15.3	Ref	
*C. trachomatis* positive, N = 62	Gardasil4^®^	21	33.9	2.21 (1.32–3.65)	**0.005**
					
*C. trachomatis* negative, N = 150	Gardasil9^®^	44	29.3	Ref	
*C. trachomatis* positive, N = 62	Gardasil9^®^	35	56.5	1.92 (1.37–2.67)	**<0.001**
					
*C. trachomatis* negative, N = 150	HPV6	8	5.3	Ref	
*C. trachomatis* positive, N = 62	HPV6	7	11.3	2.12 (0.82–5.36)	0.144
					
*C. trachomatis* negative, N = 150	HPV11	2	1.3	Ref	
*C. trachomatis* positive, N = 62	HPV11	2	3.2	2.42 (0.43–13.43)	0.582
					
*C. trachomatis* negative, N = 150	HPV16	9	6.0	Ref	
*C. trachomatis* positive, N = 62	HPV16	9	14.5	2.42 (1.03–5.63)	0.057
					
*C. trachomatis* negative, N = 150	HPV18	10	6.7	Ref	
*C. trachomatis* positive, N = 62	HPV18	7	11.3	1.69 (0.69–4.08)	0.274
					
*C. trachomatis* negative, N = 150	HPV31	9	6.0	Ref	
*C. trachomatis* positive, N = 62	HPV31	5	8.1	1.34 (0.48–3.64)	0.557
					
*C. trachomatis* negative, N = 150	HPV33	1	0.7	Ref	
*C. trachomatis* positive, N = 62	HPV33	3	4.8	7.26 (1.06–50.04)	0.076
					
*C. trachomatis* negative, N = 150	HPV45	11	7.3	Ref	
*C. trachomatis* positive, N = 62	HPV45	8	12.9	1.76 (0.75–4.02)	0.198
					
*C. trachomatis* negative, N = 150	HPV52	17	32.7	Ref	
*C. trachomatis* positive, N = 62	HPV52	2	3.2	0.28 (0.07–1.05)	0.067
					
*C. trachomatis* negative, N = 150	HPV58	15	10.0	Ref	
*C. trachomatis* positive, N = 62	HPV58	7	11.3	1.13 (0.49–2.53)	0.806
					
*C. trachomatis* negative, N = 150	HPV35	12	8.0	Ref	
*C. trachomatis* positive, N = 62	HPV35	8	12.9	1.61 (0.70–3.63)	0.304

Cervarix^®^ HPV vaccine: HPV-16 and/or -18. Gardasil^®^4 HPV types: HPV-6, -11, -16 and/or -18. Gardasil^®^9 HPV types: HPV-6, -11, -16, -18, -31, -33, -45, -52 and/or -58. The *p*-value bolded indicates the significance of the values obtained.

## Data Availability

Data is available on request from the corresponding author.

## Abbreviations

The following abbreviations are used in this manuscript:

AGYW	Adolescent girls and young women
HIV	Human Immunodeficiency Virus
HPV	Human papillomavirus
HR	High-risk
HSV	Herpes simplex virus
LR	Low risk
PCR	Polymerase chain reaction
STI	Sexually transmitted infection
WHO	World Health Organization
